# Ex-ante assessment of different vaccination-based control schedules against the peste des petits ruminants virus in sub-Saharan Africa

**DOI:** 10.1371/journal.pone.0190296

**Published:** 2018-01-19

**Authors:** Pachka Hammami, Renaud Lancelot, Joseph Domenech, Matthieu Lesnoff

**Affiliations:** 1 UMR 117 Animals, Health, Territories, Risks and Ecosystems (ASTRE), Centre de coopération internationale en recherche agronomique pour le développement (CIRAD), Campus international de Baillarguet, 34398 Montpellier, France; 2 UMR 117 ASTRE, Institut national de la recherche agronomique (INRA), Campus international de Baillarguet, 34398 Montpellier, France; 3 Consultant, La Fabrèguerie, 12170, Lédergues, France; 4 UMR Systèmes d’élevage méditerranéens et tropicaux (SELMET), CIRAD, Campus international de Baillarguet, 34398 Montpellier, France; 5 UMR SELMET, INRA, Campus international de Baillarguet, 34398 Montpellier, France; 6 UMR SELMET, Montpellier SUPAGRO, Campus international de Baillarguet, 34398 Montpellier, France; National Institute of Animal Biotechnology, INDIA

## Abstract

**Background:**

Peste des petits ruminants (PPR) is a highly contagious and widespread viral infection of small ruminants (goats and sheep), causing heavy economic losses in many developing countries. Therefore, its progressive control and global eradication by 2030 was defined as a priority by international organizations addressing animal health. The control phase of the global strategy is based on mass vaccination of small ruminant populations in endemic regions or countries. It is estimated that a 70% post-vaccination immunity rate (*PVIR*) is needed in a given epidemiological unit to prevent PPR virus spread. However, implementing mass vaccination is difficult and costly in smallholder farming systems with scattered livestock and limited facilities. Regarding this, controlling PPR is a special challenge in sub-Saharan Africa. In this study, we focused on this region to assess the effect of several variables of *PVIR* in two contrasted smallholder farming systems.

**Methods:**

Using a seasonal matrix population model of *PVIR*, we estimated its decay in goats reared in sub-humid areas, and sheep reared in semi-arid areas, over a 4-year vaccination program. Assuming immunologically naive and PPR-free epidemiological unit, we assessed the ability of different vaccination scenarios to reach the 70% *PVIR* throughout the program. The tested scenarios differed in *i)* their overall schedule, *ii)* their delivery month and *iii)* their vaccination coverage.

**Results:**

In sheep reared in semi-arid areas, the vaccination month did affect the PVIR decay though it did not in goats in humid regions. In both cases, our study highlighted i) the importance of targeting the whole eligible population at least during the two first years of the vaccination program and ii) the importance of reaching a vaccination coverage as high as 80% of this population. This study confirmed the relevance of the vaccination schedules recommended by international organizations.

## Introduction

Peste des petits ruminants (PPR) is an acute viral disease affecting goats, sheep and some wild ruminant species. It is caused by a *Morbillivirus* from the Paramyxoviridae family. The PPR virus (PPRV) is widespread in Africa, the Middle East and Asia [1]. When introduced in a fully susceptible population, it can affect up to 100% of the individuals, killing from 10 to 90% of the infected animals [2]. Therefore, the introduction of PPRV in previously free areas, as well as its endemicity in many developing countries, result in severe consequences for food security and sustainable livelihood of livestock farming communities. Such a situation exists in sub-Saharan Africa where the poor rural communities rely on small-ruminant farming and hence PPR control is of crucial importance for millions of people living in this area [3–6].

In 2015, the World Organisation for Animal Health (OIE) and the Food and Agriculture Organization of the United Nations (FAO) launched an international initiative for the progressive control of PPR and its global eradication by 2030. As a matter of fact, efficient and affordable vaccines, which provide lifelong immunity against PPRV are available [7–9]. Therefore, relying on the lessons learnt from the successful eradication of rinderpest in 2008 [10], the general PPR control strategy is based on mass vaccination of the whole small-ruminant population in endemic countries or regions [11].

The effectiveness of PPR control strategies depends on numerous factors, such as the quality of the vaccine itself, the design of vaccination campaigns (e.g., the vaccination schedule or the definition of the target population), the effectiveness of vaccine delivery (e.g., the maintenance of the cold chain or the vaccination coverage reached in the target population) or the willingness of farmers to present their animals for vaccination and to bear the cost of this vaccination [12]. However, specific data regarding PPR mass vaccination are scarce, making it difficult to plan and organize vaccination campaigns [13–15].

Following a successful pulse vaccination campaign (whole target population vaccinated within a short time period), the proportion of immunized animals in a small ruminant population (so-called post-vaccination immunity rate: *PVIR*) must be high enough to stop the PPRV transmission, thus bringing it under the epidemic threshold [16]. Because of the population turnover (offtake, mortality and birth), the immunized animals are progressively replaced with susceptible animals (newborn, purchased animals, loans) until the epidemic threshold has passed. Therefore, *PVIR* dynamics are closely related to this turnover and can be estimated using a population dynamics model [17, 18]. In a previous study, based on a predictive model of *PVIR* dynamics developed in Lesnoff et al. (2009) [17], the monthly *PVIR* dynamics was estimated for an average year following a PPR vaccination campaign in a sheep population reared in a semi-arid area [18]. In this latter study, a *PVIR* threshold of 70% was used: populations with a *PVIR* ≥ 70% were considered as protected against PPR virus transmission, according to international standards [11].

However, a wider range of agro-ecosystems is found in sub-Saharan Africa—from arid to humid environments, with contrasted small-ruminant population dynamics. In addition, a crucial step in the PPR eradication strategy is the control stage, aiming at breaking the PPRV transmission process and diminishing drastically or even suppressing the number of PPR clinical cases [11]. This control stage relies on a pluri-annual mass-vaccination schedule, associated with post-vaccination evaluation to assess the population immune status and the reduction in PPR clinical incidence.

The OIE/FAO recommended vaccination schedule involves one or two annual vaccination campaigns targeting all immunocompetent animals i.e., older than three months. These “full” campaigns may be followed by one or two annual “partial” vaccination campaigns targeting the immunocompetent offspring (age between three and 12 months), i.e. excluding the adults [11]). However, this recommendation relies more on empirical observations than on a rational assessment.

The goal of this study is to provide an *ex ante* assessment of the *PVIR* dynamics in different small ruminant smallholder farming systems, and over a pluri-annual PPR vaccination schedule.

For this purpose, we simulated the *PVIR* dynamics according to different four-year mass-vaccination schedules, in immunologically naive and PPR-free epidemiological units (Epi. U.) from contrasting agro-ecological situations: a semi-arid area where the population dynamics is highly seasonal because of climate-related nutritional constraints [19], and a sub-humid area where the population turnover is fairly constant—but faster due to higher mortality and reproduction rates [20]. As an indication of the importance of these systems, according to FAO’s database Gridded livestock of the world [21], and considering the countries located in the Sahelian region (Mauritania, Senegal, Gambia, Mali, Burkina Faso, Niger, Chad, and Sudan), more than 100 million small ruminants are reared in semi-arid or sub-humid agro-ecological areas.

For simplicity, and consistency with assumptions and findings made in Hammami et al., 2016 [18], the Epi. U. were defined as isolated populations of a few thousand small ruminants with no PPRV transmission occurring during the whole study period. As a matter of fact, in Senegal as in most Sahelian countries, agro-pastoral populations are organized in rural communities gathering several villages sharing the environment (grasslands, crop lands, water resources), and services like health centres or veterinary posts. These communities and their herds have more contacts and exchanges within them than between them. Therefore, considering this organization level as an Epi. U. makes sense in the frame of this study. At last, though PPR is endemic in this region, epidemic waves occur every two to five years [1, 22], making it relevant to consider PPR-free Epi. U., and the implementation of preventive vaccination during PPR-free years.

## Materials and methods

The Epi. U.-level monthly population dynamics was simulated using a seasonal population matrix model described in Caswell, 2001 (see chapter 13, pp. 346-369) [23]. The basic demographic model and the *PVIR* estimation method were described for one average year in Hammami et al. (2016) [18]. Following the international recommendations [11], we considered (a) different four-year vaccination schedules according to the sequence of full annual campaigns (targeting all immunocompetent animals) and partial annual campaigns (only targeting immunocompetent offspring), and (b) two possible *PVIR* variables: *i)* the vaccination month and *ii)* the vaccination coverage in the target population.

### Small ruminant farming systems

**Fig 1:**
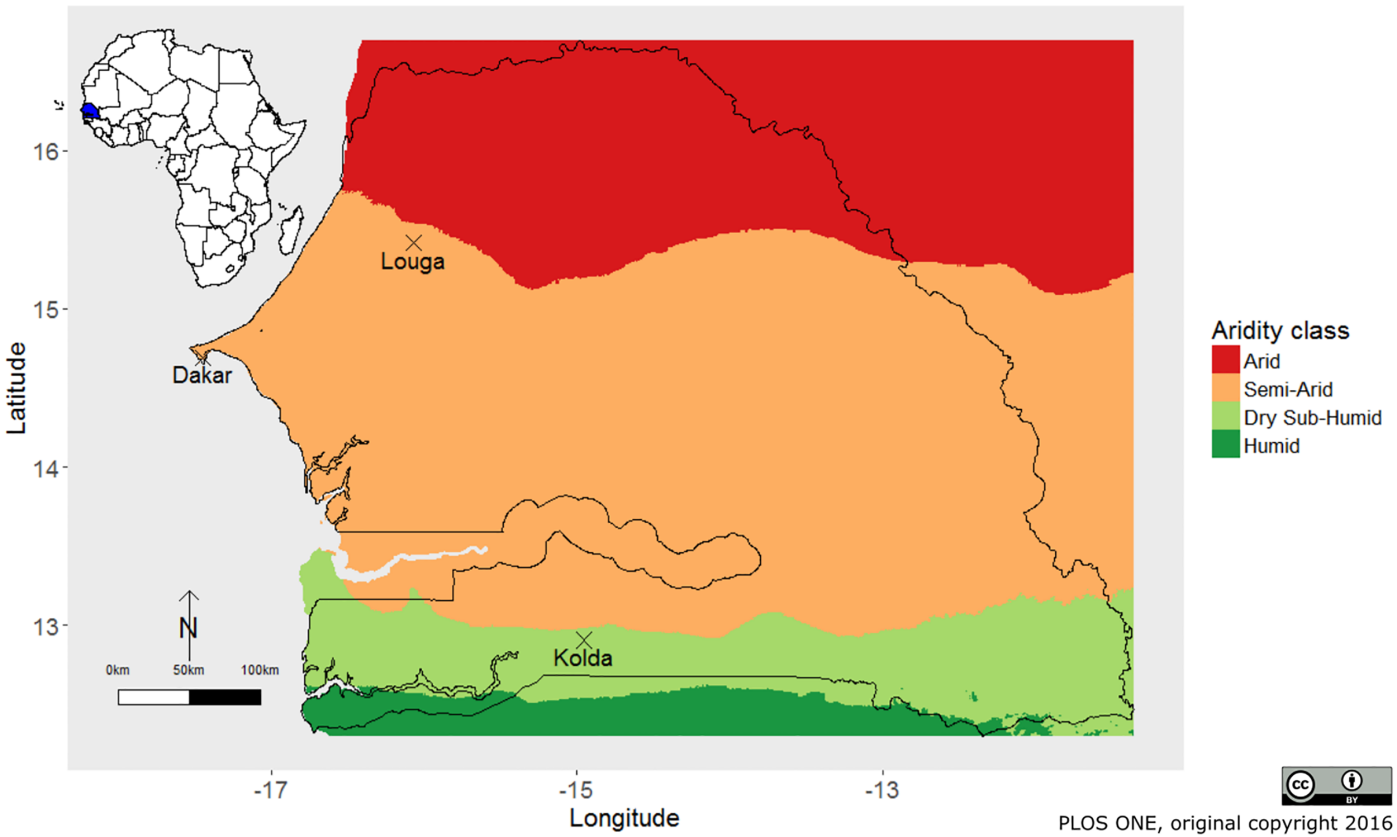
Distribution of aridity classes in Senegal. The small-ruminant follow-up demographic surveys were located in the Ndiagne municipality (Louga region) and Kolda area. This map was adapted from Hammami et al., 2016 [18] under a CC BY license, with permission from Dianne Cartwright—PLoS ONE, original copyright 2016. It was generated using data sources from Zomer et al., 2006 [25] and Trabucco et al., 2009 [26]; spatial resolution: 10 arc minutes.

To estimate the demographic parameters needed in the seasonal population matrix model, we used sheep-and-goat demographic data collected during a long-term follow-up survey implemented in smallholder farms from 1983 to 1999 [19, 20, 24]. To represent the diversity of agro-ecological situations and associated small-ruminant farming systems found in sub-Saharan Africa, we selected sheep data from the Ndiagne municipality, located in the Sahelian, semi-arid zone (Louga region, northern Senegal) and goat data from Kolda, in the Sudano-Guinean, sub-humid area of southern Senegal (Fig 1). These low-input farming systems rely on the utilization of natural grasslands. Consequently, animal breeds, as well as animal demography and productivity, are closely related to climatic and forage conditions [19, 20].

**Fig 2:**
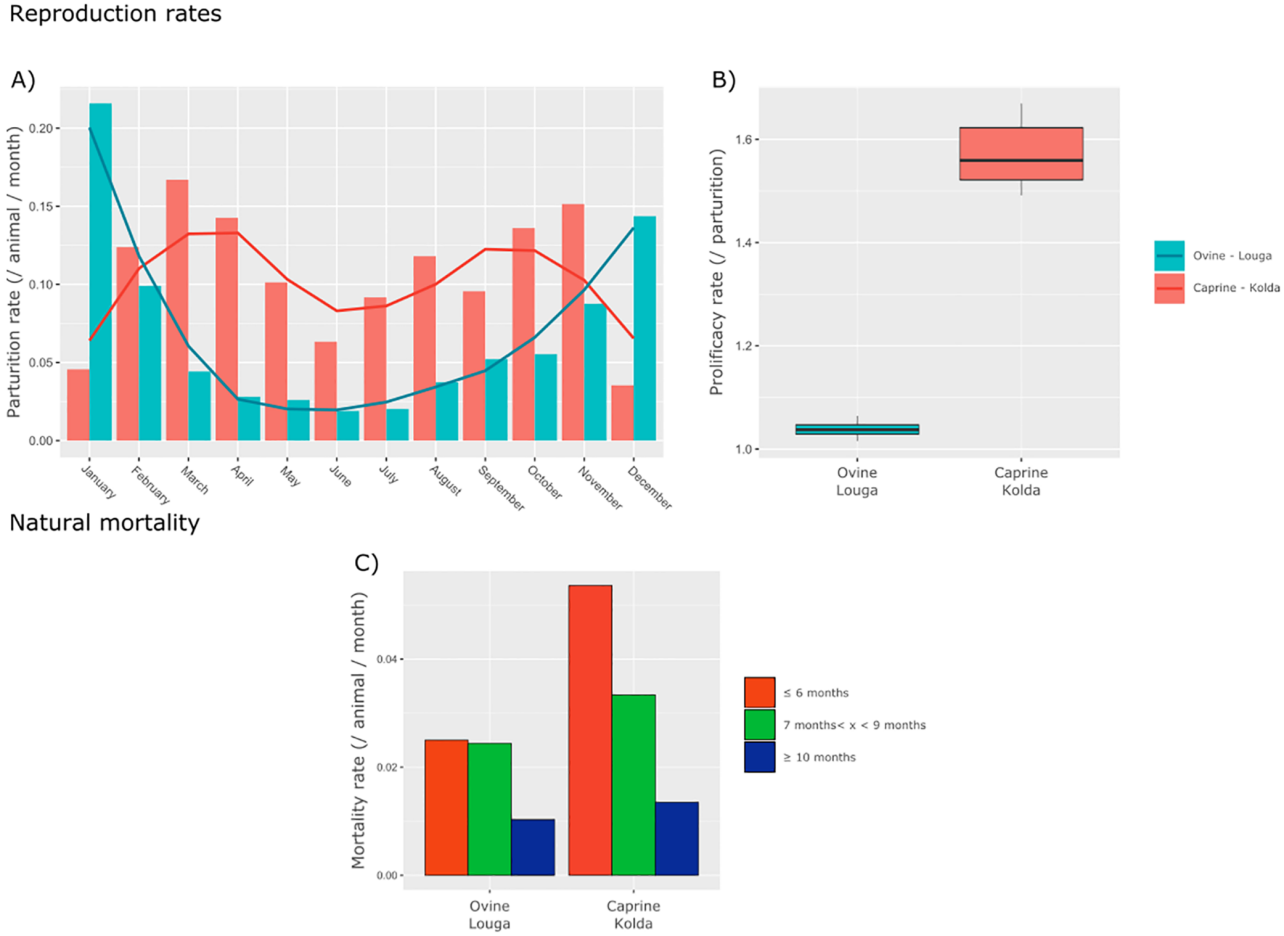
Demographic rates in Louga sheep and Kolda goats. Upper plots (A and B) show the monthly parturition rate for females older than 10 months, and the annual prolificacy rates. The lower plots show the natural mortality rates (without offtake) for three age classes.

In semi-arid areas, such as Louga region, the harsh climatic conditions experienced by the small ruminants (Sahelian breeds) during the hot, dry season result in severe constraints on their nutritional condition and physiological status. Therefore, mating is strongly seasonal, mostly occurring during the rainy season. Thus, there is a marked parturition peak between December and February [19, 27, 28] (Fig 2-A).

Conversely, in the sub-humid area of Kolda, forage resources are less scarce and available throughout the year. Consequently, mating in small ruminants (West African dwarf breeds) is less constrained than in the Sahelian zone, and parturition peaks are less marked (Fig 2-A). Also, fertility and prolificacy rates are higher, resulting in better fecundity [20, 27] (Fig 2-B). Such environment is also more suitable to parasites [29]. Therefore mortality rates are higher than in semi-arid areas (Fig 2-C). In both sites, the male offtake rate is high and strongly seasonal according to the Tabaski (Aïd El Kebir), a Muslim celebration during which a male lamb is slaughtered in most Senegalese families. However, this offtake pattern does not affect the *PVIR* dynamics [18]. Therefore, we did not account for it in this assessment.

### Design for the *PVIR* assessment

From hyper-arid to humid areas, the same general vaccination schedule is recommended for the PPR control stage [11]. In regions where PPR is endemic like in sub-Saharan Africa, it relies on the implementation of one or two successive annual mass-vaccination campaigns targeting all immunocompetent animals, i.e. sheep and goats older than three months: so-called full vaccination, hereafter denoted as “F”. These full vaccination campaigns may be complimented by one or two partial annual campaigns, hereafter denoted as “P”, targeting only the immunocompetent offspring, i.e. lambs or kids between three and 12 months.

- Whatever the schedule, in arid and semi-arid areas, a single vaccination round per annual campaign is recommended at the beginning of the dry season (from September to November) [11, 18], i.e. before the parturition peak so that newborn kids and lambs can benefit from their dam’s colostral antibodies.
- In sub-humid and humid areas, two rounds of vaccination (every six months) are recommended for each annual campaign to account for the quicker demographic turnover than in the arid and semi-arid environments [11].

The main question was the effect of different vaccination schedules (combination of full and partial vaccination campaigns) on *PVIR* during the PPR control stage. Following international recommendations, the length of this stage should range from two to five years, with an average of three years: see [11]. Therefore, we assessed the effect of one to three full annual vaccination campaigns (1F, 2F and 3F), complimented by partial annual vaccination campaigns (3P, 2P and 1P) up to a total of four years arbitrarily set as the length of the PPR control stage. The compared vaccination schedules were thus 1F3P, 2F2P, and 3F1P, for a total of four vaccination rounds in Louga (one per year), vs. eight in Kolda (two per year).

In addition, the effect of two other factors was assessed:

1. the vaccination month: following previous findings [18], three vaccination months were compared in Louga: September, October, and November. In Kolda, this factor did not affect *PVIR*, so it was not taken into account (see preliminary results in supporting information S1 Fig).
2. partial vaccination coverage, to account for possible difficulties in vaccine delivery (e.g., vaccination logistics, reluctance of farmers to bring their animals for vaccination): we assessed four vaccination coverages: 30%, 60%, 80%, and 100%.

### Assessment method

Monthly *PVIR* dynamics was simulated over a four-year control period using as input *i)* the simulated population dynamics and *ii)* the vaccination scenarios defined by the combination of vaccination schedule (1F3P, 2F2P and 3F1P), vaccination month (only in Louga: September, October and November) and vaccination coverage (30%, 60%, 80%, and 100%).

**Fig 3:**
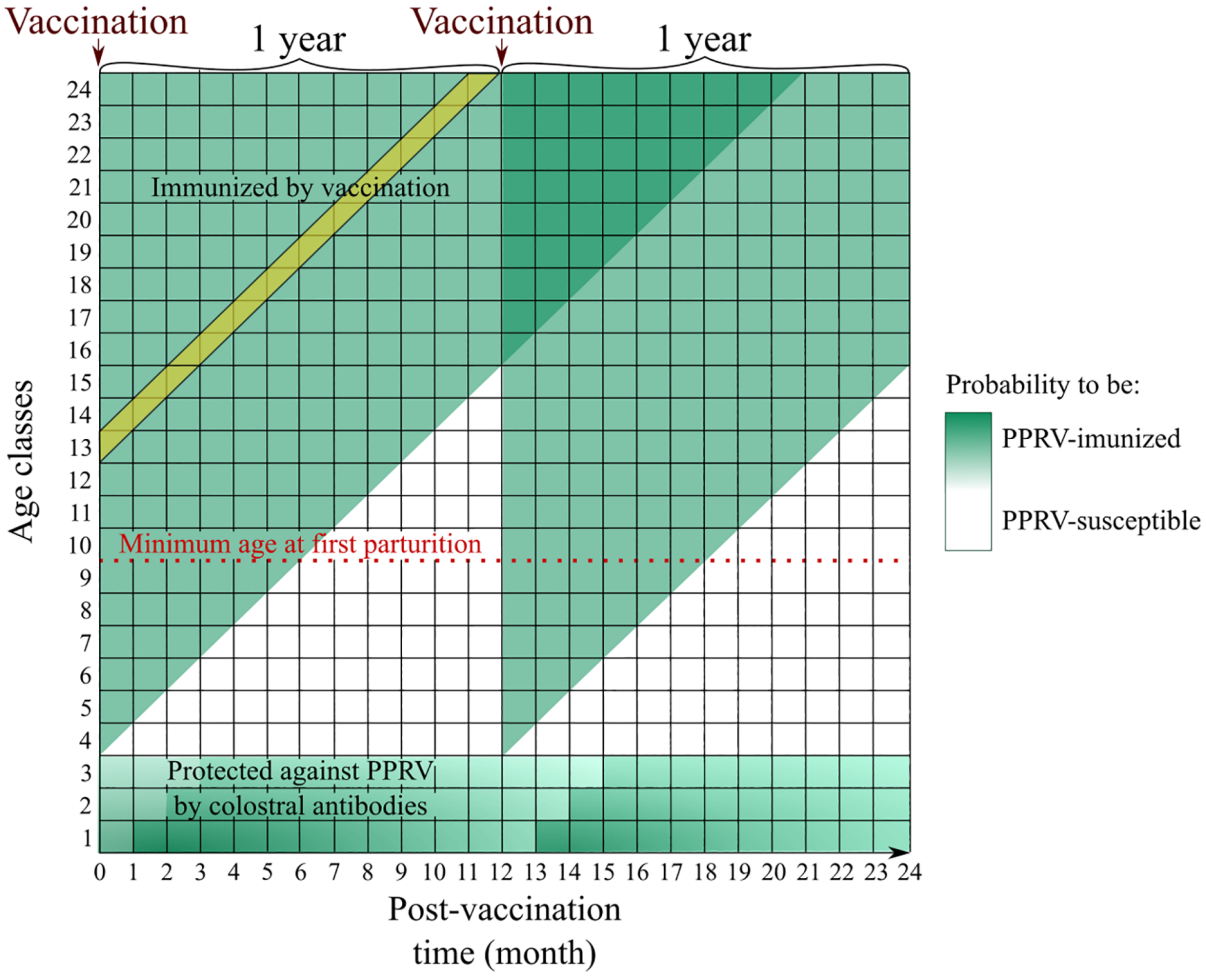
Theoretical population immunity rate dynamics over two years with annual vaccination campaigns, illustration adapted from Hammami et al., 2016 [18]. For simplicity, only animals up to two-year old were shown.

The seasonal population matrix model and the *PVIR* estimation for one average year have been previously described [18]. In this study, the *PVIR* estimation method was slightly modified to implement additional vaccination campaigns and target different subsets of the population. It was based on two main assumptions:

- for immunocompetent animals, given the lifelong immunity provided by the vaccine [7, 30], the probability for a given cohort to be immunized was constant between two vaccination rounds (a cohort represented all the animals born during the same month: see the yellow band on Fig 3 for an example);
- colostral antibodies against PPR were found in kids and lambs during the first three months of their life. This passive immunity waned and disappeared after the age of three months [31–34]. The proportion of lambs benefiting from those antibodies was proportional to dams’ immunity rate (see the shades of green in Fig 3). Moreover, the immune system of lambs under three months of age is immature and then unable to produce an efficient immune response to the vaccine inoculation [35].

Demographic rates used in the demographic model were either natural (survival, parturition and prolificacy rates), or related to livestock management practices (offtake and intake rates). Using generalized linear models, their means and standard errors were estimated for each age class (newborn, sub-adults and adults) and time step (four-month seasons: January to March, etc.) for each species and site (sheep in Louga, goats in Kolda). Assuming farmers annually targeted a constant herd size, female offtake rates were adjusted to arrive at population dynamics at the equilibrium.

To assess the uncertainty of statistical model predictions regarding the demographic parameters, 10,000 simulations were run for each scenario using random demographic rates drawn from the estimated Gaussian distribution of the demographic rates [36].

### Data analysis

In this study, we were confronted with a huge amount of data. For instance, the simulated Louga sheep dataset encompassed three vaccination schedules × three vaccination months × four vaccination coverages × 10,000 simulations = 360,000 *PVIR* dynamics, which means 360,000 × 48 months = 1,728,000 data points. In this situation, any statistical test would provide very small *p* value, whatever the actual size of the epidemiological effect [37]. Therefore, we defined an *ad-hoc* procedure to assess the epidemiological significance of the investigated parameters (vaccination schedule, vaccination month and vaccination coverage).

We defined 2F2P as the reference vaccination schedule for each site (Louga and Kolda), i.e., (i) two full annual mass-vaccination campaigns (2F) targeting the whole immunocompetent population followed by (ii) two partial annual mass-vaccination campaigns targeting the immunocompetent offspring (2P); to account for the local circumstance of small ruminant population dynamics, each annual vaccination campaign was made of one (Louga) or two (Kola) vaccination rounds. Therefore, in Louga, the reference vaccination schedule was defined by one vaccination round every 12 months, while, in Kolda, it was defined by one vaccination round every six months.

Then, we estimated the four-year *PVIR* dynamics for these two regions (Louga and Kolda), according to the other investigated variables: vaccination month and vaccination coverage.

For each combination of the variables, we computed the 50% (median), as well as the 2.5% and 97.5% (95% distribution interval) quantiles for three statistics (*θ*_*i*_) summarizing the dynamics of *PVIR*:

*T*_*thr*_ was the proportion of time during which the *PVIR* was above the 70% population immunity threshold during the four years of the PPR control stage, i.e. during which the population was protected against virus transmission. In practice, we counted the number of months during which the *PVIR* was higher than 70% and divided this number by the length of the program: 48 months. For example, if the *PVIR* remains above 70% during seven months, *T*_*thr*_ = 7/48 = 15%;
*M*_*PVIR*_ was the mean *PVIR* over the PPR control stage;
*PVIR*_48_ was the *PVIR* at the end of the PPR control stage.

We implemented the same analysis for the other investigated vaccination schedules (1F3P and 3F2P).

Thirdly, for each statistics (*θ*_*i*_ = {*T*_*thr*_, *M*_*PVIR*_, *PVIR*_48_}), we computed the relative difference (Δ_*i*,*j*_) between the reference schedule and the others for each combination of variables as:

$$\begin{array}{c} \Delta_{i,j}=\left(\theta_{i,j}-\theta_{i,\text{ref}}\right)/\theta_{i,\text{ref}} \end{array}$$

with *θ*_*i*,ref_ the statistics for the reference vaccination schedule 2F2P, and *j* the vaccination schedule to be compared with the reference schedule: 1F3P and 3F1P.

Finally, we defined an epidemiological interval around Δ_*i*_, arbitrarily set to Δ_*i*_ ± 7%. This interval represented a 5% relative difference for the 70% immunity threshold (5% ≃ 70% × 7%). The rationale of this choice is that in most PPR sero-monitoring surveys implemented to assess the post-vaccination *PVIR*, survey design and actual sample size usually provide confidence intervals of similar order: see the on-line appendix in [11].

## Results

### Overview

An overview of the results is provided in Table 1 for Louga sheep, and in Table 2 for Kolda goats.

**Table 1:** Distribution of indicators of *PVIR* according to the PPR vaccination scenarios (combination of vaccination schedule, month, and coverage (%)) for a sheep population in Louga, northern Senegal. A total of 10,000 simulations were run for each scenario. Q02.5: quantile 2.5%, Q97.5: quantile 97.5%; *T*_*thr*_: time spent above the 70% threshold; *M*_*PVIR*_: mean *PVIR* over the PPR control stage; *PVIR*_48_: *PVIR* at the end of the PPR control stage.

Schedule	Coverage	Month	*T*_*thr*_			*M*_*PVIR*_			*PVIR*_48_		
			Median	Q02.5	Q97.5	Median	Q.025	Q97.5	Median	Q02.5	Q97.5
1F3P	30	September	0	0	0	38	37	40	24	22	26
1F3P	30	October	0	0	0	37	36	39	24	22	26
1F3P	30	November	0	0	0	36	34	38	23	21	26
1F3P	60	September	8	6	8	55	53	56	39	36	41
1F3P	60	October	6	6	6	53	52	55	39	36	42
1F3P	60	November	2	2	2	51	50	53	38	35	41
1F3P	80	September	42	38	46	66	64	68	49	46	52
1F3P	80	October	35	29	38	64	62	66	49	46	52
1F3P	80	November	27	23	31	62	60	64	48	45	51
1F3P	100	September	65	62	71	77	75	79	59	55	62
1F3P	100	October	58	54	62	75	73	77	59	56	62
1F3P	100	November	50	44	54	72	70	74	57	54	61
2F2P	30	September	0	0	0	42	40	44	26	24	29
2F2P	30	October	0	0	0	41	39	43	27	24	30
2F2P	30	November	0	0	0	40	38	42	27	24	30
2F2P	60	September	21	15	25	60	58	62	42	39	45
2F2P	60	October	17	15	21	58	57	61	43	40	46
2F2P	60	November	10	8	15	57	55	59	42	39	46
2F2P	80	September	50	48	56	70	68	72	51	48	55
2F2P	80	October	46	42	50	68	66	70	52	49	55
2F2P	80	November	38	35	42	66	64	68	51	48	55
2F2P	100	September	67	65	73	79	77	80	60	56	63
2F2P	100	October	62	56	65	76	75	78	60	57	64
2F2P	100	November	54	48	56	74	72	76	59	56	63
3F1P	30	September	0	0	0	45	43	47	31	28	34
3F1P	30	October	0	0	0	44	43	47	31	28	35
3F1P	30	November	0	0	0	44	42	46	32	28	35
3F1P	60	September	33	27	40	63	61	65	47	43	50
3F1P	60	October	27	23	33	62	60	64	47	44	51
3F1P	60	November	21	15	25	60	58	62	47	44	51
3F1P	80	September	56	54	62	72	71	74	55	51	58
3F1P	80	October	52	46	54	70	69	72	55	52	59
3F1P	80	November	44	40	48	69	67	71	55	52	59
3F1P	100	September	67	67	75	80	78	81	61	58	64
3F1P	100	October	65	58	67	78	76	79	62	59	65
3F1P	100	November	56	50	58	76	74	77	62	58	65

**Table 2:** Distribution of indicators of *PVIR* according to the PPR vaccination scenarios (combination of vaccination schedule and coverage (%)) for a goat population in Kolda, southern Senegal. A total of 10,000 simulations were run for each scenario. Q02.5: quantile 2.5%, Q97.5: quantile 97.5%; *T*_*thr*_: time spent above the 70% threshold; *M*_*PVIR*_: mean *PVIR* over the PPR control stage; *PVIR*_48_: *PVIR* at the end of the PPR control stage.

Schedule	Coverage	*T*_*thr*_			*M*_*PVIR*_			*PVIR*_48_		
		Median	Q02.5	Q97.5	Median	Q02.5	Q97.5	Median	Q02.5	Q97.5
1F3P	30	0	0	0	33	33	34	29	28	30
1F3P	60	4	2	6	59	59	60	52	50	53
1F3P	80	56	52	60	72	72	73	63	62	65
1F3P	100	100	100	100	82	82	83	72	70	74
2F2P	30	0	0	0	40	39	40	33	32	34
2F2P	60	25	21	27	64	63	64	55	53	56
2F2P	80	65	60	69	74	74	75	65	63	66
2F2P	100	100	100	100	82	82	83	72	70	74
3F1P	30	0	0	0	43	43	44	39	38	40
3F1P	60	33	29	38	66	65	66	58	56	59
3F1P	80	69	65	73	75	74	76	66	64	68
3F1P	100	100	100	100	82	82	83	72	70	74

In Louga sheep, a vaccination coverage of 30% never allowed reaching, or getting close to, the 70% population immunity threshold, whatever the vaccination month and vaccination schedule: indeed, the cumulative effect of successive vaccination campaigns on *PVIR* was not high enough to compensate the sheep population turnover. A vaccination coverage of 60% did not allow reaching this threshold with the 1F3P schedule; however, the median value for the estimated mean *PVIR* (*M*_*PVIR*_) was close to, or above, 60% for 2F2P and 3F1P. Regarding the immunity rate at the end of the control stage (*PVIR*_48_), it was close to, or above, 50% irrespective of the vaccination schedule and vaccination month. Logically, better values were obtained with 80% and 100% vaccination coverages. However, even with the 3F1P schedule and a full vaccination coverage, the median value for *M*_*PVIR*_ was never greater than 80%, and the median value for *PVIR*_48_ just exceeded 60%.

Regarding the vaccination month, the best values for the investigated indicators were obtained in September, with the exception of *PVIR*_48_ for which the highest median values were reached in October and November. However the differences in *PVIR*_48_ between the three vaccination months were low, without any epidemiological consequence.

In Kolda goats, similar trends were observed: a 30% vaccination coverage never allowed reaching values close to the targets for any of the three indicators, with the same result as with Louga sheep regarding the lack of cumulative effect along the control stage irrespective of the vaccination schedule. As soon as the vaccination coverage was 60% or higher, median values greater than 60% were reached for *M*_*PVIR*_ and *PVIR*_48_. However, vaccination coverages ≥ 80% and at least two full vaccination campaigns were needed to reach values of *T*_*thr*_ > 60%.

*In fine*, a vaccination coverage of 30% or lower was certainly insufficient to get an appropriate *PVIR*, whatever the agro-ecological zone and vaccination schedule. Also, a single full vaccination campaign (1F3P) only provided correct *PVIR* for vaccination coverages of at least 80%.

### The reference vaccination schedule 2F2P

**Fig 4:**
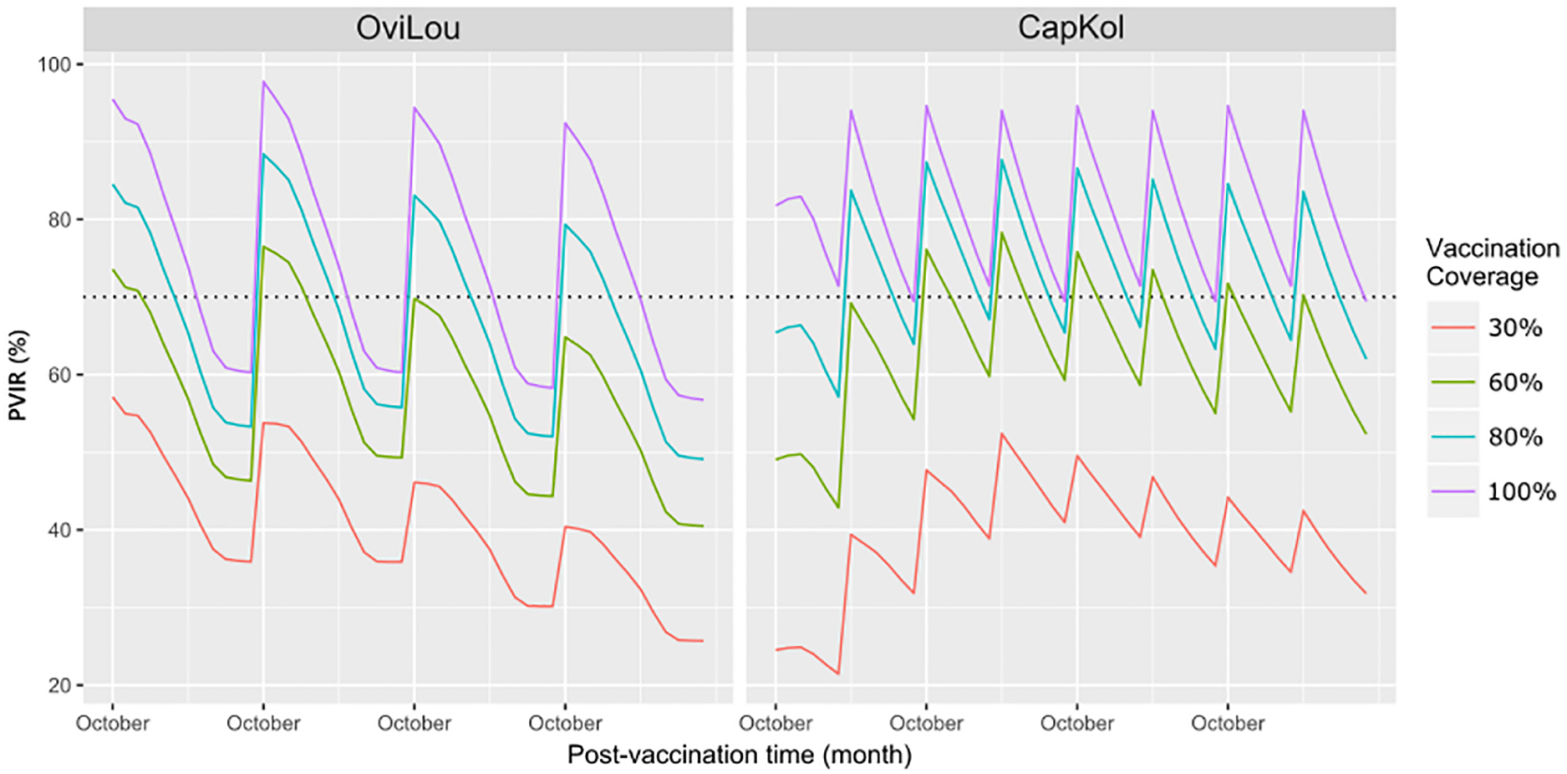
*PVIR* dynamics during a four-year control stage (2F2P schedule) in a sheep population from Louga (northern Senegal: Red line) and in a goat population from Kolda (southern Senegal: Blue line), with different vaccination coverages and the first vaccination round implemented in October. The vaccination coverages represent the proportion of vaccinated animals among the immunocompetent animals (> 3 months). Each line represents the *PVIR* for the whole population. The 70% *PVIR* threshold is represented by a black dotted line.

In both investigated agro-ecological systems, with a full vaccination coverage (100% fo the immunocompetent population), the 2F2P vaccination schedule allowed keeping the *PVIR* above, or close to, the 70% population immunity threshold during the PPR control stage (Fig 4). In Louga sheep (OviLou: a single vaccination round each year), the *PVIR* was above this threshold 67% of the time, declining below it eight months after each vaccination round. In Kolda goats (CapKol: two vaccination rounds each year), the *PVIR* was always above the threshold during the control stage. However, the *PVIR* estimated immediately after the vaccination round was higher in OviLou (96%, 95% confidence interval [95; 96]) than in CapKol (81% [80; 82]).

With vaccination coverages of > 80%, these *PVIR* results should allow reaching the goals assigned to the PPR control stage: break PPRV transmission and suppress PPR clinical expression. Therefore, the data support the relevance of the 2F2P vaccination schedule as a reference for comparisons with other schedules.

### Relative difference with respect to the 2F2P vaccination schedule

**Fig 5:**
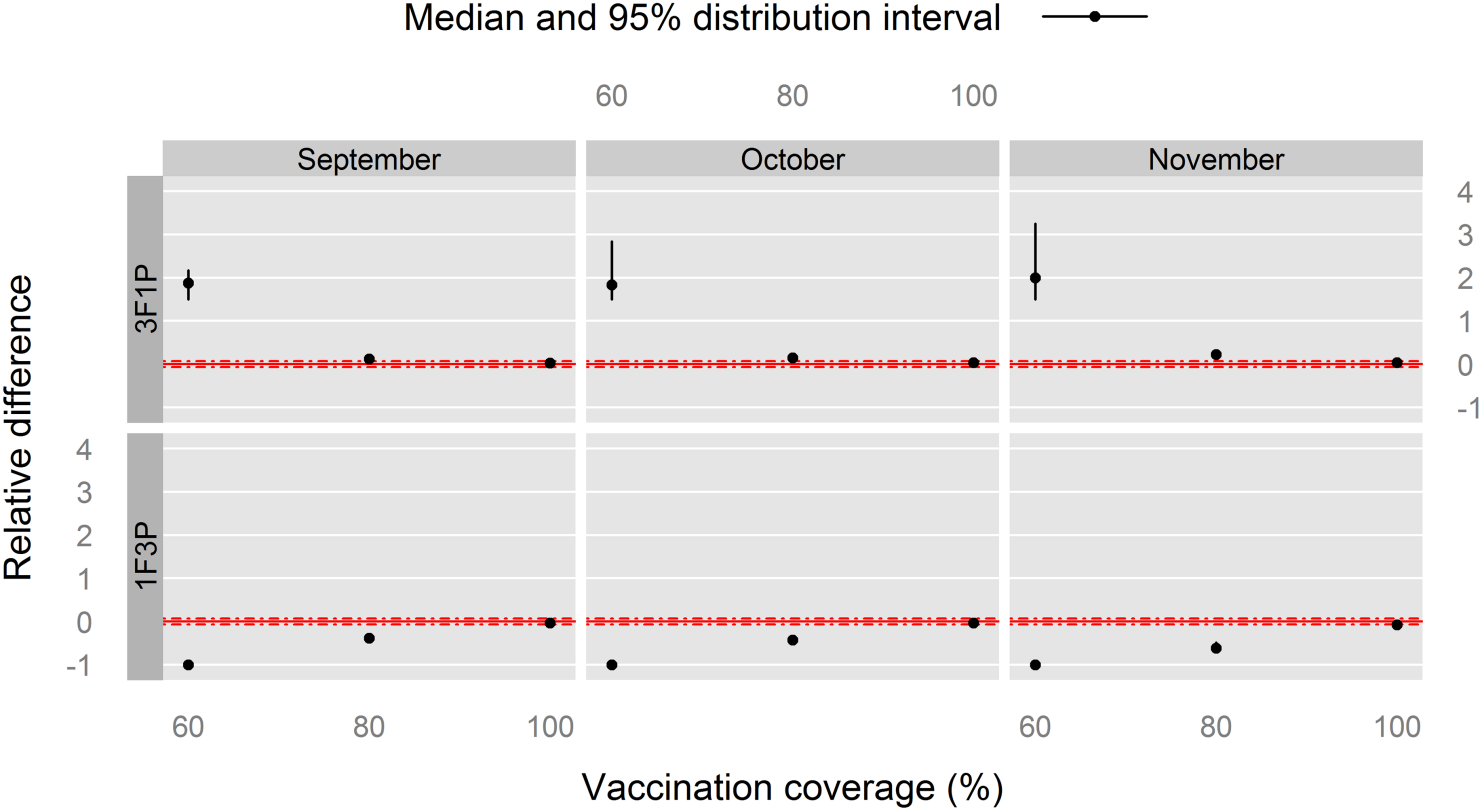
Relative difference in time spent above the 70% threshold (*T*_*thr*_) for post-vaccination *PVIR* with respect to the 2F2P vaccination schedule for Louga sheep, northern Senegal. The red, solid line indicates the reference situation (2F2P), and the red, dashed lines above and under it indicate a positive or negative relative 7%-difference with this reference situation.

**Fig 6:**
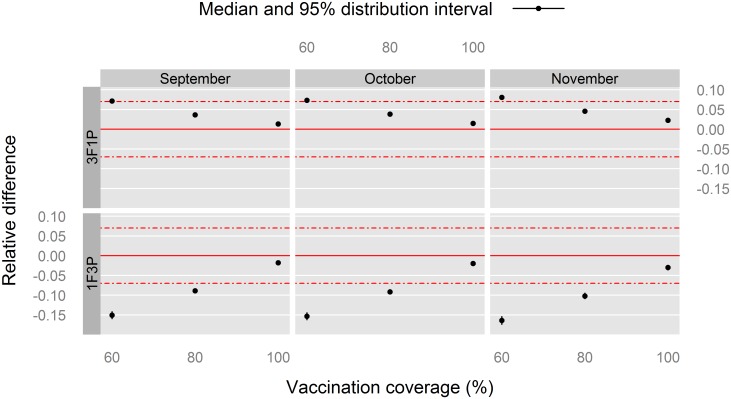
Relative difference in the mean *PVIR* (*M*_*PVIR*_) with respect to the 2F2P vaccination schedule for Louga sheep, northern Senegal. The red, solid line indicates the reference situation (2F2P), and the red, dashed lines above and under it indicate a positive or negative relative 7%-difference with this reference situation.

**Fig 7:**
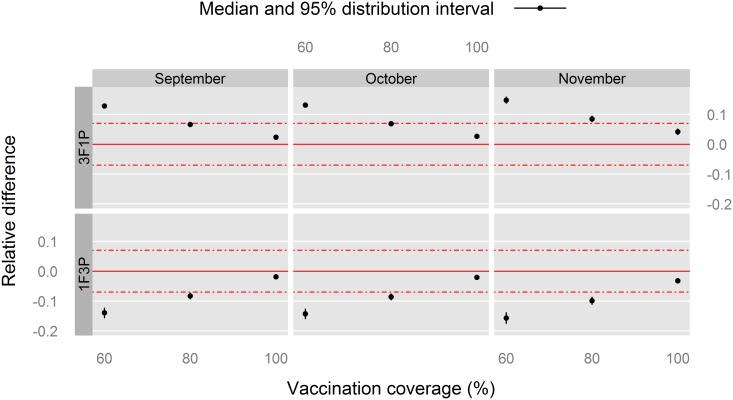
Relative difference in the *PVIR* at the end of the PPR control stage (*PVIR*_48_) with respect to the 2F2P vaccination schedule for Louga sheep, northern Senegal. The red, solid line indicates the reference situation (2F2P), and the red, dashed lines above and under it indicate a positive or negative relative 7%-difference with the reference situation.

In Louga sheep, the combined effects of the vaccination month and the vaccination coverage is shown on Figs 5 to 7. A common pattern is the low effect of the vaccination month. We did not investigate this effect further.

For the time spent with *PVIR* ≥ 70% (*T*_*thr*_, Fig 5), the 60% vaccination coverage provided a positive relative difference for the 3F1P vaccination schedule (upper panel of plots) with a substantial epidemiological meaning, in favour of 3F1P. This can be seen as a cumulative effect along the PPR control stage, made possible by three successive full vaccination campaigns. This effect disappeared with higher vaccination coverage: the immunity rate was close to its maximum value after each annual vaccination campaign, thus nullifying the possibility of a cumulative effect.

Not surprisingly, a symmetrical negative relative difference was observed for the 1F3P vaccination schedule (lower panel of plots), indicating that this schedule was consistently worse than the 2F2P schedule in terms of *PVIR*, with the exception of the 100% vaccination coverage, for which the relative difference was not epidemiologically important.

For the relative difference in the mean *PVIR* (*M*_*PVIR*_, Fig 6), all the values fell within the 7% epidemiological interval with the exception of the relative difference for the 60% vaccination coverage with the 1F3P vaccination schedule. We can conclude that for this indicator, there was no important epidemiological difference between the 2F2P and 3F1P vaccination schedules when vaccination coverage was ≥ 80%.

The same observations applied to the statistics of the *PVIR* at the end of the PPR control stage (*PVIR*_48_, Fig 7).

To sum up the findings for Louga sheep, the 60% vaccination coverage consistently provided worse relative indicators than 80% and 100% coverages, supporting the implementation of three full vaccination campaigns rather than one or two. On the other hand, with the latter coverages, no marked difference was observed between the 1F3P and 3F1P vaccination schedules, with respect to 2F2P.

**Fig 8:**
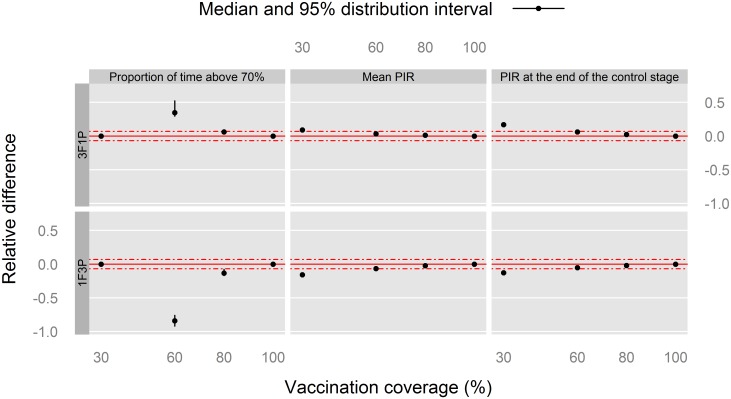
Relative difference in indicators of post-vaccination *PVIR* with respect to the 2F2P vaccination schedule for Kolda goats, southern Senegal. The red, solid line indicates the reference situation (2F2P), and the red, dashed lines above and under it indicate a positive or negative relative 7%-difference with the reference situation.

Regarding the Kolda goats (Fig 8), similar conclusions can be made. A cumulative effect of successive vaccination campaigns was observed for the relative difference in *T*_*thr*_ with the 30% as well as 60% vaccination coverages. On the other hand, no marked difference was observed between the 1F3P and 3F1P vaccination schedules, with respect to 2F2P, for the two other indicators (*M*_*PVIR*_ and *PVIR*_48_) with vaccination coverages ≥ 60%.

## Discussion

### Validity of the small ruminant demographic dataset

Demographic data were collected in smallholder, low-input small ruminant farming systems, the most challenging systems for PPR vaccination, because herds are small, sparse, mobile and therefore difficult to reach and monitor. These systems rely on the availability of natural resources: surface water during the rainy season, grasslands, and crop by-products (in the southern regions). Small ruminant demography (mortality, age at first parturition, fecundity) and growth strongly depend on these conditions [28, 38]. However, when these conditions are better (higher rainfall), more room is given by the farmers to crop production, whereas ruminant livestock farming is left to more arid environment. Therefore, though such climate changes occurred in Senegal and in the whole Sahel with increased rainfall and vegetation index [39], the demographic parameters of small ruminants reared in low-input smallholder farming systems probably remained stable. Obviously, formal evidences would be better than assumptions, but the dataset we have used for this paper has no up-to-date counterpart. Thus, this study highlights the need for implementing more long-term follow-up demographic studies in small ruminant population to increase the accuracy of such demographic models.

### Vaccination month

The effectiveness of a vaccination campaign is directly related to the actual vaccination coverage. If the population is mainly composed of immunocompetent animals at the time of vaccination, the *PVIR* is higher than if the vaccination campaign is achieved just after the parturition peak (when present) [18]. Indeed, newborn animals benefit from maternal antibodies (passive immunity). This immunity wanes after the age of three months, and offspring thus become susceptible to the virus. However, their immune system is then mature enough to produce antibodies [33, 40].

Therefore, when the small ruminant reproduction shows a marked seasonal pattern, like in arid and semi-arid areas, the ideal period for implementing the vaccination would be three months after the parturition peak, i.e. between April and June in the case of Louga [18, 27, 28]. However, this period is also the beginning of the hot, dry season. At that time, farmers lack financial resources to pay for the vaccination because they already sold their crops [19, 41]. Moreover, sheep and goats are left straying at that time, and farmers—as well as vaccinators, are reluctant to catch and gather them when air temperature often exceeds 40°C. At last, most animals are in a poor body condition, thus possibly affecting their immune system and causing vaccination failure at the individual level, even if the vaccine was correctly administered [42, 43]. Therefore, in arid and semi-arid areas, the best period to implement PPR vaccination should be from September (end of the rainy season, when body condition is optimal) and November (before the parturition peak, to make sure that dams may transmit colostral antibodies to their offspring). Estimates of the median indicators (Table 1: *T*_*thr*_, *M*_*PVIR*_, and *PVIR*_48_) consistently showed that September was the best month to implement vaccination. This trend was not altered by the vaccination coverage (Figs 5 to 7).

### Vaccination coverage and vaccination schedule

Three major features were highlighted by our results:

- Vaccination coverage must be > 60% to reach the 70% *PVIR* threshold (*M*_*PVIR*_ indicator), whatever the vaccination month (Louga sheep) and site (Louga vs. Kolda). However in Kolda goats, the 3F1P schedule associated with a 60% vaccination coverage brought *M*_*PVIR*_ very close to the threshold, in the context of two vaccination rounds per annual campaign.
- When the vaccination coverage decreased, the number of full vaccination campaigns had to be higher to reach the 70% *PVIR* threshold.
- Symmetrically, when the number of full vaccination campaigns was higher, the *PVIR* were better, whatever the vaccination month (Louga) and *PVIR* indicator.

Good results were obtained in Kolda with the 1F3P schedule and 60% vaccination coverage: *M*_*PVIR*_ and *PVIR*_48_ were both above the 70% threshold. This is certainly related to the fact that two vaccination rounds were achieved each year. However, it highlights the possible vaccine savings that could be done to compensate the higher vaccination frequency in sub-humid and humid areas. Nevertheless, several pitfalls are encountered with partial vaccination:

- vaccine cost is a minor part of the overall vaccination costs encompassing, among other things, wages of veterinary staff, vehicles, gasoline and maintenance of the cold chain. For instance, in a study of vaccination costs for the rinderpest campaign, Ly et al. (1998) reported vaccine represented 23% of the overall costs [44].
- Partial vaccination is not easy to implement in actual field conditions: indeed, given the general lack of vaccination pens for small ruminants, kids and lambs have to be sorted and caught one by one by the farmers and vaccinators, thus causing additional work and decreasing the productivity of vaccination teams.
- Farmers might be reluctant to only vaccinate the offspring, thus neglecting the adult animals—in particular ewes and nannies, which constitute their productive capital.

Therefore, in practice, we would recommend to implement only full vaccination campaigns.

### The *PVIR* threshold

The *PVIR* threshold is defined as the population immunity level needed to break the PPRV transmission cycle and to suppress the PPR clinical signs during the PPR control stage [11]. Setting the *PVIR* to 70% is a somewhat arbitrary, because estimating it at the national level from theoretical considerations—or using numerical simulations, is a difficult task (see below). In this study, we made considerable simplifications by limiting the assessment to small, homogeneous, and PPR-free Epi. U. Some milestones are useful to help assessing the threshold itself.

Firstly, this PVIR threshold assumption is related to *T*, the control effort to bring the pathogen transmission below the epidemic threshold. In homogeneous populations, *T* = 1 − 1/*R*_0_, where *R*_0_ is the basic reproduction number, i.e. the number of secondary disease cases after the introduction of as single infectious individual in a fully susceptible population [16]. Therefore, in our simple epidemiological framework, it is crucial to get accurate *R*_0_ estimates to derive adequate values for *T*. Unfortunately, very few empirical estimates (obtained during PPR outbreaks in field situations) are available [45, 46]. These estimates provided very high values for *T* (> 80%) but they were obtained in conditions rather different from those encountered in sub-Saharan Africa (animal breeds and density, farming systems, etc.). Furthermore, to our knowledge, no PPRV transmission model has been published so far. Therefore, it is critical to implement systematic PPR outbreak investigation studies, and to promote mathematical modelling work to get reliable and adapted estimates of *T*.

Regarding this latter topic, coupling the *PVIR* model with a dynamic PPRV transmission model might allow more precise estimations of the *PVIR* threshold needed to remain below the epidemic threshold [16]. However, building such a model is a complex task even starting with PPRV transmission in a small Epi. U. under the assumption of homogeneous mixing. Indeed, at last two host species need to be taken into account (sheep and goats) because they are both present in the same herds and villages, with frequent contacts and thus many PPRV transmission opportunities. These species and breeds have specific susceptibilities to PPRV, clinical expression and mortality rates. For instance, West-African dwarf breeds (found in Kolda) are much more susceptible than Sahelian breeds (found in Louga) [1]. Moreover, basic knowledge is scarce regarding important epidemiological parameters, such as the excretion duration of viable PPRV in body fluids and feces [47–49], or the susceptibility of the different small ruminant species and breeds found in sub-Saharan Africa [1]. In addition, PPRV virulence shows some variability [50]. With so many uncertainties, we believe our *PVIR*-based approach is useful and less prone to problematic assumptions.

Secondly, higher *T* values (80%) were set in the case of rinderpest control. This threshold was never reached during decades when mass-vaccination campaigns were organized by the Joint Program 15 (JP15), Pan-African rinderpest campaign (PARC), and Pan-African control of epizootics (PACE) eradication programs [10, 51, 52]. Even in a small country like Senegal, with a limited cattle population, only the oldest age classes were close to it after a decade of mass-vaccination campaigns [53]. Nevertheless, rinderpest was finally eradicated, even though the last virus sanctuary was located in remote and unsafe places [54] where the vaccination coverage could not be very high. This might be an indication that for the control stage, the threshold—while useful and important to maintain the motivation of stakeholders, was probably an overestimate of the actual *T* value, at least for sub-Saharan Africa.

Finally, Morocco was confronted for the first time to the emergence of PPR in 2008. National veterinary services immediately implemented a PPR control strategy based on a 3F1P schedule, followed by a successful eradication stage. During the control stage, more than 80% of the national small ruminant stock (> 20 million heads) was vaccinated each year: pulse vaccination, a single vaccination round per year. In 2012, a nationwide serological survey was implemented and provided an estimate of 70% for *PVIR*_48_ in adult ewes [55]. The overall *PVIR*_48_ was probably lower than 70%. This is an empirical evidence that in small ruminant farming systems similar to those found in Morocco (mixture of smallholder sheep farms and fattening lots), the actual threshold might even be lower than 70%.

There is a good consistence between the results obtained in this simulation study and the assumptions done when preparing the PPR global control and eradication strategy (GCES) [11] which was designed with the objectives to control and then eradicate PPR at the country, region and global levels, particularly with reference to the choice of the vaccination protocols.

The FAO-OIE PPR GCES uses vaccination as the major tool for combating PPR in endemic countries. Nevertheless, other methods and tools must not be forgotten [56]. Disease surveillance, including field and laboratory work, as well as preparedness for early action to eliminate new PPR outbreaks occurring in free regions or countries.

Together with an appropriate communication strategy, the successful implementation of the GCES also relies on the quality of the animal health services provided to the farmers, particularly by the private and public veterinary services (VS) as well as by other local health stakeholders at the producer levels including community animal health workers [57].

In many sub-Saharan countries, VS efficiency and effectiveness with respect to the farmers must be improved. This is why one of the three components of the FAO-OIE PPR GCES is devoted to strengthening VS in charge of preventing and controlling animal diseases.

## Conclusion

This study provided evidences that PPR control should be possible in sub-Saharan Africa. It looks safer to promote the implementation of at least two full mass-vaccination campaigns (2F schedule). In sub-humid and humid areas, partial vaccination (offspring) might provide good epidemiological results, but its practical interest is questionable. Whatever the vaccination schedule and agro-ecological area, the most important feature remains the vaccination coverage and for this purpose, a key aspect is the correct identification of the efficacy of the local socio-technical networks actually providing animal health services to the farmers and especially the quality of VS.

This study was limited to a single, PPR-free Epi. U. The real world is much more complex, with intense livestock movements (trade, transhumance) possibly associated with the introduction of non-immunized animals, or even pathogen agents [58, 59]. Therefore, it is important to maintain the vaccination coverage (introduced animals should be immunized), and to prevent the introduction of the virus once the vaccination has ceased. Thus, the role of the Veterinary Services is crucial both in designing / implementing the vaccination campaigns—together with other stakeholders, and also in other aspects of PPR control, such as recording and accounting for animal mobility. In turn, this information might be used to improve the design of national PPR control program, with a regional and international coordination.

## Supporting information

S1 Fig Annual dynamics of post-vaccination *PIR* in goat herds, Kolda (Senegal), assuming full vaccination coverage (*p* = 1). A total of 144 vaccination scenarios are represented crossing the Tabaski month (12 plots) with the vaccination month (12 lines). On each plot, the origin of the *x* axis is the vaccination month. The horizontal dotted line represents the 80% protective threshold.
(TIFF)

S1 Table Raw data set with individual follow-up in sheep herds of Louga (Senegal). The file structure and its use are fully described in [60].
(ZIP)

S2 Table Raw data set with individual follow-up in goat herds of Kolda (Senegal). The file structure and its use are fully described in [60].
(ZIP)
