# Supplementary figures and images for: Ex-ante assessment of different vaccination-based control schedules against the peste des petits ruminants virus in sub-Saharan Africa
Source: PLoS One. 2018 Jan 19;13(1):e0190296. doi: 10.1371/journal.pone.0190296 (PMC5774693; doi:10.1371/journal.pone.0190296)

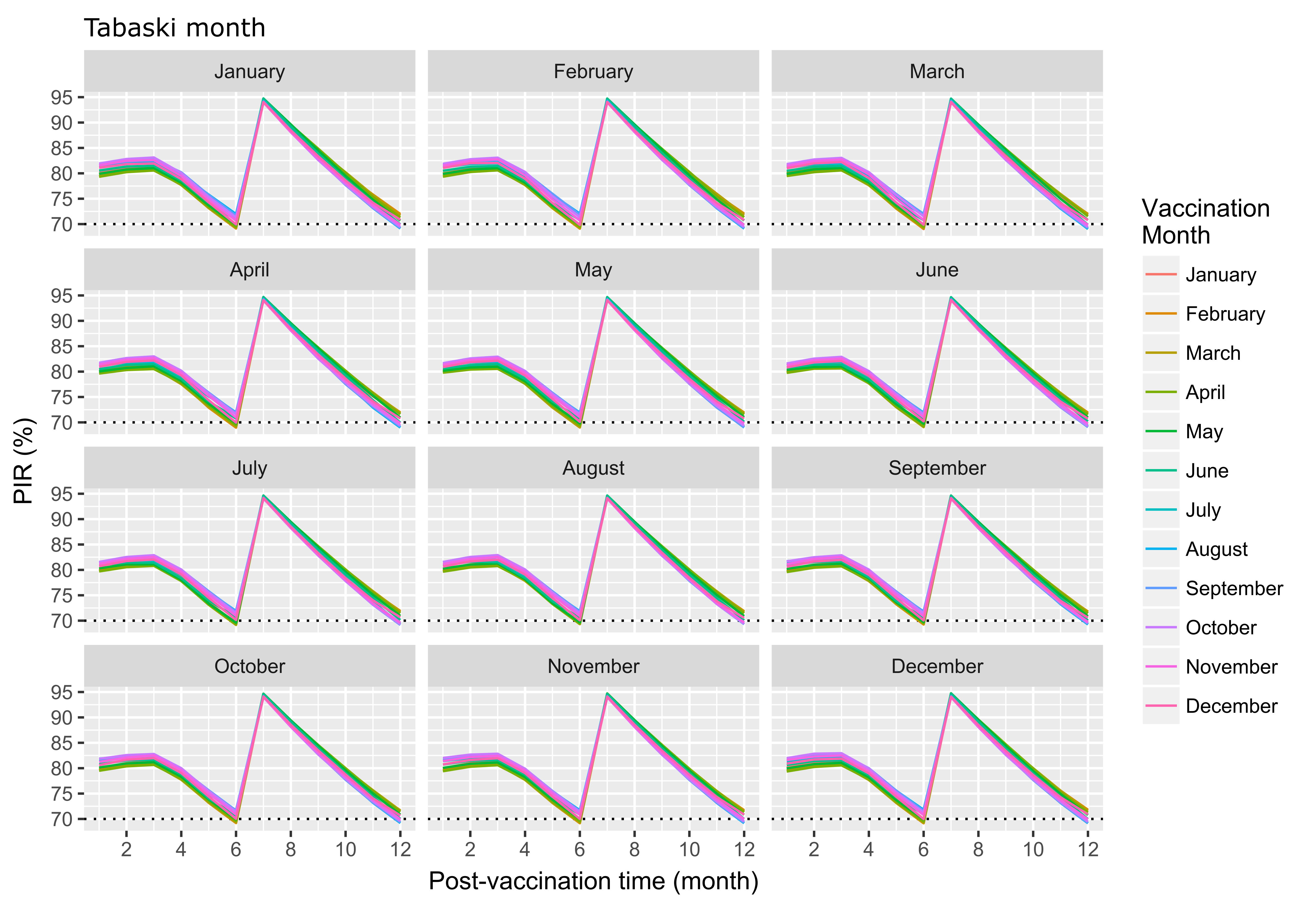

Supplement: S1 Fig — A total of 144 vaccination scenarios are represented crossing the Tabaski month (12 plots) with the vaccination month (12 lines). On each plot, the origin of the x axis is the vaccination month. The horizontal dotted line represents the 80% protective threshold. (TIFF) [file pone.0190296.s001.tiff]
